# Rebuilding DEMATEL threshold value: an example of a food and beverage information system

**DOI:** 10.1186/s40064-016-3083-7

**Published:** 2016-08-22

**Authors:** Yi-Fang Hsieh, Yu-Cheng Lee, Shao-Bin Lin

**Affiliations:** 1 Department of Food and Beverage Management, Taipei College of Maritime Technology, No. 212, Sec. 9, Yanping N. Rd., Shilin Dist., Taipei City, 111 Taiwan, ROC; 2 Graduate Institute of Technology Management, Chung Hua University, 707, Sec. 2, WuFu Rd., Hsinchu, 30012 Taiwan, ROC; 3 Department of Finance and Banking, Shih Chien University, No. 70, Dazhi St., Zhongshan Dist., Taipei City, 104 Taiwan, ROC

**Keywords:** Decision-making trial and evaluation laboratory (DEMATEL), Threshold value, Fractional factorial design, Decomposed theory of planned behavior model (DTPB model), Food and beverage information system

## Abstract

This study demonstrates how a decision-making trial and evaluation laboratory (DEMATEL) threshold value can be quickly and reasonably determined in the process of combining DEMATEL and decomposed theory of planned behavior (DTPB) models. Models are combined to identify the key factors of a complex problem. This paper presents a case study of a food and beverage information system as an example. The analysis of the example indicates that, given direct and indirect relationships among variables, if a traditional DTPB model only simulates the effects of the variables without considering that the variables will affect the original cause-and-effect relationships among the variables, then the original DTPB model variables cannot represent a complete relationship. For the food and beverage example, a DEMATEL method was employed to reconstruct a DTPB model and, more importantly, to calculate reasonable DEMATEL threshold value for determining additional relationships of variables in the original DTPB model. This study is method-oriented, and the depth of investigation into any individual case is limited. Therefore, the methods proposed in various fields of study should ideally be used to identify deeper and more practical implications.

## Background

The decision-making trial and evaluation laboratory (DEMATEL) method can be applied to solve complicated problems. It operates mainly through collection of experts’ opinions by viewing the degree of influence between elements, the use of matrix operations to obtain a causal relationship between the elements, and the establishment of similar structural equation modeling network diagrams. The core DEMATEL method comprises four calculation steps: (1) define the scale; (2) build a direct-relation matrix; (3) calculate a normalized matrix; (4) calculate a direct/indirect relationship matrix *T*. The threshold value is set after Step (4). The setting of a threshold value is typically influenced by problem complexity and divergent expert opinions.

Some researchers use various methods to set up the threshold value, whereas some ignore explanations about the threshold value setting (Li and Tzeng 2009; Hu et al. 2011; Lee et al. 2013). However, an overly high threshold value inappropriately reduces the significance of expert opinions and oversimplifies the problem, whereas an exceedingly low threshold value results in divergent opinions and a lack of focus. Therefore, if a threshold value cannot appropriately differentiate expert opinions, it cannot accurately present the critical factors of a complex problem.

To determine a conventional threshold value purely using expert opinions or researcher judgments and to prevent inappropriate threshold value from affecting the definitions of problems, some scholars studied the setting of DEMATEL threshold value. For instance, Li and Tzeng (2009) proposed a maximum mean de-entropy algorithms (MMDE) to determine threshold value. MMDE was mainly used to decide whether a node is suitable to express in the impact-relations map. However, in the past, operating with subjective expert opinions, DEMATEL was unable to find appropriate threshold value. Even though some scholars proposed the MMDE method, that method did not alleviate the problem of computational complexity.

Therefore, the study proposes a type of simple and reasonable method to set threshold value. The concept of fractional factorial design was expected to enable scientific DEMATEL threshold value and to avoid subjective DEMATEL threshold value.

The present author is currently teaching university classes about dining information systems. In addition to a food service worker’s typical professional skills, a crucial skill valued by the food service job market is the ability to think systematically and to control work-related information flows to maximize efficiency. The introduction of food and beverage information system can greatly improve the quality of a food and beverage service. However, the improvement in service quality triggered by the information system depends heavily on whether the workers make the most of the system. In this study, the decomposed theory of planned behavior (DTPB) proposed by Taylor and Todd (1995) is adopted to examine the behaviors and inclinations of dining service workers in using a food and beverage information system. A new method is proposed to determine DEMATEL threshold value and to explain the behaviors and inclinations of dining service workers in using the food and beverage information system.

This paper discusses the importance of the reasonable calculation of DEMATEL threshold value using the example of a food and beverage system. Subsequently, the DTPB information model theory that is used in this study is described. The proposed calculation steps and fractional factorial designs provide a reasonable and quick way to calculate DEMATEL threshold value. A food and beverage information system is planned by combining DEMATEL and DTPB model to discover the behaviors and inclinations of dining service workers in using the food and beverage information system. This paper argues for conclusions and notes limitations of the present work.

## Literature review

### Theory of planned behavior

**Fig. 1:**
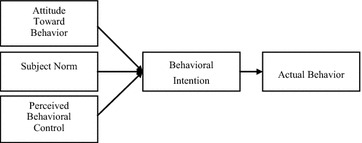
Theory of planned behavior model

In the theory of reasoned action (TRA), an individual behavior proceeds from free will and an individual can completely determine whether to execute a behavior (Fishbein and Ajzen 1975). However, apart from situations of free will, the expression of some behaviors also requires the coordination of resources and opportunities during execution of those behaviors; for example, whether an individual possesses abilities for behavioral control and implementation can affect his or her behavioral intention (BI); and individual ability to control this is called perceived behavioral control (PBC). Therefore, Ajzen (1985) revised the TRA by adding PBC. Ajzen held that when predicting BI, one can delve into behavioral attitudes and subjective norms (SNs), but whether an individual has the opportunities and resources to execute the behaviors in question and whether the individual is able to control these behaviors, affects BI; this theory is the theory of planned behavior (TPB). Its framework is shown in Fig. 1.

### Decomposed theory of planned behavior

Taylor and Todd (1995) proposed the DTPB model to explain human behavior regarding information technology. DTPB model was founded on the original TPB and Technology Acceptance Model (TAM). DTPB adds creative characteristics in order to establish three aspects that influence behaviors and inclinations, namely attitude, SN, and PBC. Their study indicated that the predictions of DTPB model were slightly more accurate than TAM and TPB. DTPB model had more explanatory power. This can be explained as follows:

1. Actual behavior: This is an individual’s intention to perform a behavior which is a function of attitude toward behavior, subjective norms, and PBC.
2. BI: BI refers to the tendency of individuals to engage in some particular behavior.
3. Attitude: Attitude refers to the individual performance of specific acts held positive or negative rating
4. SN: SN refers to an individual when the performance of a particular behavior, that affect them essential concerns, social pressure to support or not.
5. PBC: PBC refers to the degree of personal performance when a particular behavior, self-control resources.

Taylor and Todd (1995) wrote that attitude can be derived from the perceived characteristics of an innovation. Three characteristics of information technology acceptance and use are relative advantage, complexity, and compatibility (Moore and Benbasat 1991). Relative advantage refers to the benefits of innovative practices relative to the original level. Complexity refers to difficulties in the understanding, learning, and awareness of the innovative technology.

Taylor and Todd (1995) wrote that the definition of relative advantage and complexity are similar to the ideas of perceived usefulness (PU) and perceived ease of use (PEU) in the TAM model. Compatibility refers to innovation in line with the current value of potential recipient, the extent of past experience, and current needs. To the notions of PU and PEU can be added the notion of compatibility. Attitude can be expressed as the following three variables (Rogers 1983; Davis 1989):

(6) PU: the subjective belief of the user that the use of a particular information technology will increase the level of his or her job performance.
(7) PEU: the subjective belief of the user that the use of the Information Technology investment will not require significant effort and energy.
(8) Compatibility: this is the perception of an individual that the innovative behaviors adopted match previous experience, current value, and needs; the more compatible the innovation is, the more chance it has of being adopted.

**Fig. 2:**
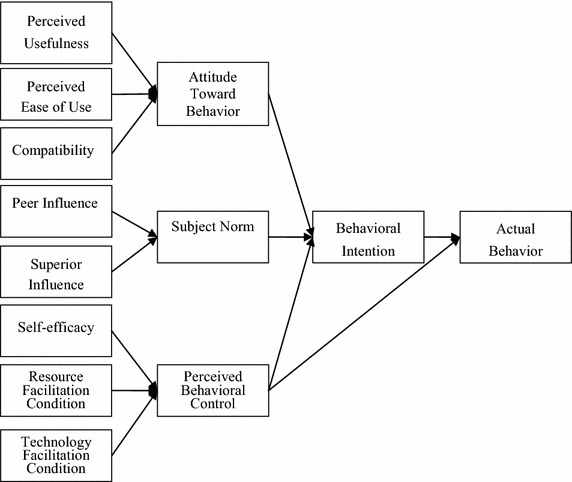
Decomposed theory of planned behavior model

In terms of SNs, Taylor and Todd (1995) pointed out three kinds of referent groups, peers, superiors, and subordinates. In this study, SN can be broken into the following two variables:

(9) Peer influence: when an individual is engaged in a certain behavior, positive inputs from his or her peers, such as friends and coworkers, increase the probability that he or she continues the behavior.
(10) Superior influence: this means that positive inputs from a worker’s supervisor regarding a behavior make it more likely that the worker continues the behavior.
(11) PBC is divided into the following three variables (Bandura 1977):
(12) Self-efficacy: this means that when an individual perceives that he or she is capable of a certain behavior, it is more likely that he or she engages in that particular behavior.
(13) Resource facilitating conditions: these refer to the availability of the resources needed to facilitate a behavior when an individual is engaged in this behavior. The resources can be time, money, equipment, and so on.
(14) Technological facilitating conditions: these mean that when an individual believes that he or she has sufficient time, money, equipment, or other resources for a certain behavior as well as the technical capability of engaging in such a behavior, it is more likely that he or she executes the behavior. The framework is shown in Fig. 2.

Use of the DTPB model has several advantages. First, we can understand the different facets of antecedents in the DTPB model (Bagozzi 1981; Shimp and Kavas 1984). Second, because of DTPB’s decomposed structure, the relationships between the various factors and facets are clear and easy to understand, and therefore DTPB model can explain the factors that may affect actual use (Mathieson 1991).

In previous DTPB model studies, structural equation modeling was used to analyze the relationships between variables (Shih and Fang 2004; Lin 2007; Malek et al. 2010). However, accurate analysis was difficult because incorrect conclusions were often caused by some variables that did not satisfy the assumption of independence. To solve this, Lee et al. (2013) employed the expert-opinion-oriented DEMATEL to reestablish the causal relationships between DTPB variables and their mutual influences. Despite the efforts to reestablish the causal relationships between DTPB variables and their mutual influences using the DEMATEL method, this method was dependent on expert opinions regarding the degrees of influence between elements. In particular, a clear definition of threshold value was still missing in the DEMATEL method.

## DEMATEL threshold value

DEMATEL was built by the Battelle Geneva Institute to solve difficult problems (Gabus and Fontela 1973; Fontela and Gabus 1976). It was intended to find direct and indirect relationships, and to gauge strength of influence between different elements in the complex environment.

Recently, the DEMATEL has been widely introduced to identify key factors in complicated problems. For instance, Wang et al. (2016) sought to identify the key barriers to the implementation of green supply chain management in the packaging industry by using DEMATEL. Asad et al. (2016) attempted to study the key factors affecting customer satisfaction in an internet banking system so that bank operations might be prioritized to reflect cause and effect relationships. Pan and Ngnyen (2015) proposed an approach for helping manufacturing companies identify the key performance evaluation criteria for achieving customer satisfaction through balanced scorecard (BSC) and multiple criteria decision-making (MCDM) approaches. Uygun et al. (2015) integrated DEMATEL and fuzzy ANP techniques for evaluation and selection of outsourcing providers for a telecommunication company. Lu et al. (2013) improved RFID adoption in Taiwan’s healthcare industry using a DEMATEL technique with a hybrid MCDM model. Lee et al. (2010) applied fuzzy DEMATEL to the TAM to verify benefits. These DEMATEL-related studies suggest that this approach has been extensively adopted in various fields of study and widely accepted.

Briefly, the procedure of DEMATEL can be implemented as follows:

Step 1: Define the evaluation scale
  Define the evaluation scale to show the degree of impact. Values on the 10-point scale represent degrees of influence from “no influence” to “great influence”.
Step 2: Build a direct-relation matrix
  A direct-relation matrix *X* is produced by integrating the opinions of experts, where *x*_*ij*_ expresses the extent to which $x_{i}$ affects $x_{j}$; the value of any element on the diagonal is 0.
  1 $$ X = \left[ {\begin{array}{*{20}c} 0 &\quad {x_{12} } &\quad \cdots &\quad {x_{1n} } \\ {x_{21} } & \quad 0 & \quad\cdots & \quad{x_{2n} } \\ \vdots & \quad\vdots & \quad\ddots & \quad\vdots \\ {x_{n1} } & \quad{x_{n2} } & \quad\cdots & \quad  0 \\ \end{array} } \right] $$
Step 3: Normalize the direct-relation matrix
  A direct-relation matrix is normalized with matrix *X*, using the following method:
  2 $$ {\text{Define}}\;\lambda = \frac{1}{{\begin{array}{*{20}c} {Max} \\ {1 \le i \le n} \\ \end{array} \left( {\sum\nolimits_{j = 1}^{n} {x_{ij} } } \right)}}\quad {\text{and}}\quad N = \lambda X $$
Step 4: Calculate a direct/indirect relationship matrix *T*
  Because the normalized matrix *N* is known, the following equation can produce the total matrix *T*:
  3 $$ T = \mathop {\lim }\limits_{k \to \infty } \left( {N + N^{2} + \cdots + N^{k} } \right) = N\left( {I - N} \right)^{ - 1} $$
  where *I* is an identity matrix.

Fractional factorial design is typically applied in experiments for developing new products and improving existing production methods. The success of such experiments depends on factor configuration before the experiment and effect analysis after the experiment. To reduce experimental cost, time, and complexity, it is crucial that no significant factors be excluded. Numerous studies have addressed this problem, most of which have adopted the effect-sparsity assumption proposed by Box and Meyer (1986). The effect-sparsity assumption is that among the various effects, only a few are significant. Regarding this assumption, several scholars have written that significant effects can be treated as outliers, which are cut off from samples, and no outlier effects can be adopted for estimation of experimental errors (Lenth 1989; Schneider et al. 1993; Haaland and O’Connell 1995).

Generally, when an experimental design involves numerous factors, a screening experiment should be conducted first, in which crucial factors that exert effects on response variables are discovered. The crucial factors can then be selected to undergo an optimization experiment for determining their optimal input levels. However, because of limited experimental resources, unreplicated factorial design is typically adopted in screening experiments and no significant effects are eliminated. Consequently, when the data of such experiments are analyzed with no degree of freedom left for estimating experimental errors, traditional *t* tests and F tests cannot be adopted to determine the significance of effects. To solve this problem, several scholars have proposed various analytical methods. Daniel (1959) was the first to investigate this problem, and numerous scholars have developed distinct statistical methods based on the fractional factorial design to identify which effects are influential. Among these scholars, Lenth (1989) proposed the effect-sparsity assumption, based on the research of Box and Meyer (1986). This assumption indicates that only a few factorial effects have specific influences on response variables. Therefore, a censoring approach and pseudostandard errors are employed to estimate the standard deviations of effects; these can lead to statistics similar to those of *t* tests. The threshold value from this method are then adopted to determine effect significance. Because the calculations required for the method proposed by Lenth are relatively simple, this method is widely applied in unreplicated factorial designs for analyzing test data.

Based on the effect-sparsity assumption, the method proposed by Lenth (1989) estimates $\tau$ by assuming that the median of $\left| {\hat{\beta }_{k} } \right|$ equals $\frac{2}{3}\tau$ when ${\text{H}}_{0} :\beta_{1} = \cdots = \beta_{m} = 0$. Initially, because $\frac{median}{1 \le k \le m}\left| {\hat{\beta }_{k} } \right| \approx 0.67\tau$ the initial estimate of $\tau$ is defined as $S_{0} = 1.5 \times \frac{median}{1 \le k \le m}\left| {\hat{\beta }_{k} } \right|$. Subsequently, because $P_{r} = \left[ {\left| {\hat{\beta }_{k} } \right| \ge 2.5\tau |\left| {\beta_{1} = \cdots = \beta_{m} = 0} \right| \approx 0.01} \right]$, Lenth considered that estimating $\tau$ using the $\left| {\hat{\beta }_{k} } \right|$ value that are smaller than 2.5*S*_0_ should generate relatively robust estimates. Consequently, Lenth defined pseudostandard error (PSE) as ${\text{PSE}} = 1.5 \times \frac{median}{{\left| {\hat{\beta }_{k} } \right| < 2.5S_{0} }}\left| {\hat{\beta }_{k} } \right|$ where $\frac{median}{{\left| {\hat{\beta }_{k} } \right| < 2.5S_{0} }}\left| {\hat{\beta }_{k} } \right|$ denotes the median generated from the absolute regression coefficients that are smaller than 2.5$S_{0}$. In other words, PSE represents the $S_{0}$ established after the regression coefficients that are possible active effects have been deleted. Subsequently, Lenth defined the margin of error (ME) of various regression coefficients as ${\text{ME}} = t_{{1 - \frac{\alpha }{2};\frac{m}{3}}} \times {\text{PSE}}$ and adopted ME value to test effect significance. In the equation, $t_{{1 - \frac{\alpha }{2};\frac{m}{3}}}$ represents the quartile of $\left[ {1 - \frac{\alpha }{2}} \right]$ in a *t* distribution where the degree of freedom is $\frac{m}{3}$. Finally, Lenth suggested that the effects corresponding to the absolute regression coefficients that are less than or equal to ME value should be regarded as nonsignificant. The calculation steps of Lenth’s method are as follows:

Step 1: Calculate $S_{0}$ the initial value of $\tau$
  4 $$ S_{0} = 1.5 \times \frac{median}{1 \le k \le m}\left| {\hat{\beta }_{k} } \right| $$
Step 2: Calculate PSE
  5 $$ {\text{PSE}} = 1.5 \times \frac{median}{{\left| {\hat{\beta }_{k} } \right| < 2.5S_{0} }}\left| {\hat{\beta }_{k} } \right| $$
Step 3: Calculate ME
  6 $$ {\text{ME}} = t_{{1 - \frac{\alpha }{2};\frac{m}{3}}} \times {\text{PSE}} $$

DEMATEL threshold value is set based on Lenth’s principles of distinguishing effect significance, whereby threshold value and ME are adopted to eliminate nonsignificant factors for obtaining factors with significant influences in scenarios with complex problems or factors. When Lenth’s method is combined with the DEMATEL method, suitable threshold value can be determined by calculating ME value, and problems resulting from inappropriate DEMATEL threshold value can be effectively resolved.

## Example: food and beverage information system in DTPB model

### Research design

This objective of this study is to demonstrate how the DEMATEL threshold value can be quickly and reasonably determined by combining DEMATEL and DTPB models to identify the key factors in a complex problem. A food and beverage information system is presented as an example. The combination of DEMATEL and DTPB models as applied to the food and beverage information system were analyzed to discover the behaviors and inclinations of dining service workers regarding use of the food and beverage information system. These findings should contribute to the further introduction of food and beverage information systems and the improvement of food and beverage service quality.

In this study, a fractional factorial design was employed to build DEMATEL threshold value to obtain critical factors of a complex system. Invitations were issued to 20 experts, who were asked to share their insights on the use of a DEMATEL-DTPB combination for the analysis of worker behaviors relevant to a food and beverage information system. These experts, who answered the questionnaires developed for this study, included restaurant owners, waiters who have direct contact with customers, and college faculty members who teach the theory of food and beverage information systems. The questionnaire survey was administered between October and December of 2015. There were more males than females among these 20 experts. More than half of the experts had a college degree or a postgraduate degree. The majority of the experts were between 40 and 50 years of age. The survey included face-to-face interviews with the experts. The questions provided in the questionnaire were based on food and beverage information systems. The interviewees were asked to estimate the degree of influence on the variables of the DTPB model based on their knowledge regarding the system. A 10-point scale was introduced to rate the degree of influence from “no influence” to “great influence.”

The original DTPB model has 13 variables: PU (A_1_), PEU (A_2_), compatibility (A_3_), peer influence (A_4_), superior influence (A_5_), self-efficacy (A_6_), resource facilitation conditions (A_7_), technology facilitation conditions (A_8_), attitude toward behavior (A_9_), SN (A_10_), PBC (A_11_), BI (A_12_), and actual behavior (A_13_).

### Data analysis

Based on the analysis procedures of DEMATEL, a direct relationship matrix *X* was first established, based on the opinions of the aforementioned 20 professionals, to adopt the mean and establish a direct relationship matrix *X* according to Eq. (1), which is shown in Table 1.

**Table 1:** Direct relationship matrix of food and beverage information system in DTPB model

*X*	A_1_	A_2_	A_3_	A_4_	A_5_	A_6_	A_7_	A_8_	A_9_	A_10_	A_11_	A_12_	A_13_
A_1_	0	0	0	1	2	1	1	1	9	1	1	1	1
A_2_	8	0	0	1	1	1	1	1	10	1	1	1	1
A_3_	0	0	0	1	1	1	1	1	9	1	1	1	1
A_4_	0	0	0	0	0	1	1	1	1	9	1	1	1
A_5_	0	0	0	0	0	1	1	1	1	9	1	1	1
A_6_	1	1	1	1	1	0	1	1	1	1	10	1	1
A_7_	1	1	1	1	1	1	0	1	1	1	8	1	1
A_8_	1	1	1	1	1	1	1	0	1	1	7	1	1
A_9_	1	1	0	0	0	3	0	0	0	0	0	8	1
A_10_	0	0	0	0	0	0	2	2	0	0	0	8	1
A_11_	5	5	0	0	0	5	0	0	0	0	0	9	1
A_12_	0	0	1	0	0	1	1	1	1	0	0	0	10
A_13_	1	1	1	1	2	1	1	1	1	1	1	1	0

In Eq. (2), a normalized direct-relation matrix, wherein the sum of the row vector was used as the normalized basis, produced the value 1/(8 + 1+1 + 1+1 + 1+10 + 1+1 + 1+1) = 1/27. The normalized direct-relation matrix is shown in Table 2.

**Table 2:** Normalized matrix of food and beverage information system in DTPB model

*N*	A_1_	A_2_	A_3_	A_4_	A_5_	A_6_	A_7_	A_8_	A_9_	A_10_	A_11_	A_12_	A_13_
A_1_	0.000	0.000	0.000	0.037	0.074	0.037	0.037	0.037	0.333	0.037	0.037	0.037	0.037
A_2_	0.296	0.000	0.000	0.037	0.037	0.037	0.037	0.037	0.370	0.037	0.037	0.037	0.037
A_3_	0.000	0.000	0.000	0.037	0.037	0.037	0.037	0.037	0.333	0.037	0.037	0.037	0.037
A_4_	0.000	0.000	0.000	0.000	0.000	0.037	0.037	0.037	0.037	0.333	0.037	0.037	0.037
A_5_	0.000	0.000	0.000	0.000	0.000	0.037	0.037	0.037	0.037	0.333	0.037	0.037	0.037
A_6_	0.037	0.037	0.037	0.037	0.037	0.000	0.037	0.037	0.037	0.037	0.370	0.037	0.037
A_7_	0.037	0.037	0.037	0.037	0.037	0.037	0.000	0.037	0.037	0.037	0.296	0.037	0.037
A_8_	0.037	0.037	0.037	0.037	0.037	0.037	0.037	0.000	0.037	0.037	0.259	0.037	0.037
A_9_	0.037	0.037	0.000	0.000	0.000	0.111	0.000	0.000	0.000	0.000	0.000	0.296	0.037
A_10_	0.000	0.000	0.000	0.000	0.000	0.000	0.074	0.074	0.000	0.000	0.000	0.296	0.037
A_11_	0.185	0.185	0.000	0.000	0.000	0.185	0.000	0.000	0.000	0.000	0.000	0.333	0.037
A_12_	0.000	0.000	0.037	0.000	0.000	0.037	0.037	0.037	0.037	0.000	0.000	0.000	0.370
A_13_	0.037	0.037	0.037	0.037	0.074	0.037	0.037	0.037	0.037	0.037	0.037	0.037	0.000

After normalization, the direct/indirect relationship matrix *T* was derived using Eq. (3), as shown in Table 3.

**Table 3:** Direct/indirect matrix of food and beverage information system in DTPB model

*T*	A_1_	A_2_	A_3_	A_4_	A_5_	A_6_	A_7_	A_8_	A_9_	A_10_	A_11_	A_12_	A_13_
A_1_	0.084	0.065	0.030	0.063	0.110	0.152	0.086	0.086	0.432	0.121	0.161	0.287	0.195
A_2_	0.407	0.085	0.039	0.082	0.106	0.200	0.109	0.109	0.597	0.144	0.208	0.377	0.254
A_3_	0.082	0.063	0.029	0.062	0.072	0.148	0.082	0.082	0.427	0.107	0.156	0.277	0.188
A_4_	0.062	0.048	0.027	0.022	0.031	0.106	0.095	0.095	0.113	0.374	0.142	0.253	0.172
A_5_	0.062	0.048	0.027	0.022	0.031	0.106	0.095	0.095	0.113	0.374	0.142	0.253	0.172
A_6_	0.216	0.167	0.074	0.077	0.094	0.178	0.103	0.103	0.239	0.134	0.526	0.369	0.245
A_7_	0.191	0.148	0.070	0.073	0.089	0.190	0.062	0.097	0.218	0.127	0.440	0.328	0.222
A_8_	0.179	0.138	0.068	0.071	0.086	0.179	0.094	0.059	0.208	0.124	0.396	0.308	0.210
A_9_	0.104	0.080	0.033	0.026	0.038	0.181	0.046	0.046	0.111	0.048	0.111	0.409	0.219
A_10_	0.052	0.040	0.033	0.023	0.033	0.064	0.113	0.113	0.078	0.042	0.101	0.390	0.207
A_11_	0.343	0.265	0.052	0.055	0.079	0.324	0.085	0.085	0.286	0.099	0.209	0.577	0.320
A_12_	0.069	0.053	0.069	0.034	0.053	0.111	0.081	0.081	0.137	0.063	0.113	0.134	0.452
A_13_	0.108	0.083	0.058	0.060	0.106	0.113	0.079	0.079	0.151	0.116	0.142	0.193	0.112

A more obvious cause-and-effect relationship was then determined. The values of the direct/indirect relationship matrix table were set by a threshold value.

Initially, a threshold value was calculated using Eq. (4). The median (0.108) was selected from the direct/indirect relationship matrix *T*. The initial value of $\tau$ (*S*_0_) was calculated as follows:

$$ S_{0} = 1.5 \times 0.108 = 0.162. $$

Using Eq. (5), after the values in the direct/indirect relationship matrix *T* that were greater than or equal to 2.5$S_{0}$ had been deleted, the median (0.103) was obtained as ${\text{PSE}} = 1.5 \times 0.103 = 0.1545$.

Finally, using Eq. (6), given α = 0.05 and df = 56, it was calculated that $t_{{1 - \frac{\alpha }{2};\frac{m }{3}}}$ = 2.0033 and ${\text{ME}} = 2.0033 \times 0.1545 = 0.310$. An effect level lower than 0.310 was treated as a relationship that was not causal. A relationship matrix with a significant effect was determined, as shown in Table 4.

**Table 4:** Significant direct/indirect matrix of food and beverage information system in DTPB model (rebuild threshold value = 0.310)

*T*	A_1_	A_2_	A_3_	A_4_	A_5_	A_6_	A_7_	A_8_	A_9_	A_10_	A_11_	A_12_	A_13_
A_1_									0.432				
A_2_	0.407								0.597			0.377	
A_3_									0.427				
A_4_										0.374			
A_5_										0.374			
A_6_											0.526	0.369	
A_7_											0.440	0.328	
A_8_											0.396		
A_9_												0.409	
A_10_												0.390	
A_11_	0.343					0.324						0.577	0.320
A_12_													0.452
A_13_													

**Fig. 3:**
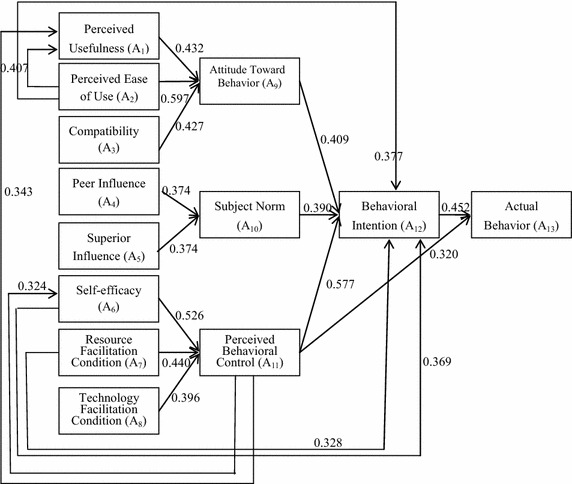
New DTPB model constructed by a DEMATEL method

Table 4 not only gives the degrees of influence among the variables of the DTPB model after the integration of the DEMATEL and DTPB models, but also helps clarify the new relationships among the variables that are apparent after the rebuilding of DTPB model using the DEMATEL model combined with the analysis results with the original DTPB model. For example, some new relationships between variables were apparent, as shown in Fig. 3. The influence coefficients of A_1_, A_2_, and A_3_ on A_9_ are 0.432, 0.597, and 0.427, respectively; the influence coefficients of A_4_ and A_5_ on A_10_ are 0.374 and 0.374, respectively; and those of A_6_, A_7_, and A_8_ on A_11_ are 0.526, 0.440, and 0.396, respectively. The influence coefficients of A_9_, A_10_, and A_11_ on A_12_ are 0.409, 0.390, and 0.577, respectively; the influence coefficients of A_11_ and A_12_ on A_13_ are 0.320 and 0.452, respectively.

Some new relationships between variables can be described as follows. For instance, A_2_ not only affected A_9_, but also exerted additional effects on A_1_ and A_12_; the effect coefficients were 0.407 and 0.377, A_6_ and A_7_ not only affected A_11_, but had new relationships on A_12_ with effect coefficients of 0.369 and 0.328, respectively. A_11_ not only affected A_12_ and A_13_, but had new relationships with A_1_ and A_6_ with effect coefficients of 0.343 and 0.324, respectively.

## Conclusions and limitations

DEMATEL can assist in locating the core problem and improving complex systems through the degrees of interrelationship among quantified quality attributes. However, DEMATEL threshold value is often set by experts according to their own judgments. If not established reasonably, the thresholds will affect the causal relationships between variables. To discover the critical attributes of a complex problem, threshold value must be adequate for further analysis.

A simple and quick method was proposed in this paper for determining DEMATEL threshold value by adopting the concepts of fractional factorial design. A food and beverage information system was analyzed with a DTPB model; that model with rebuilt with DEMATEL threshold value. The results proved that by rebuilding, one can calculate reasonable DEMATEL threshold value and one can determine additional relationships of variables from the original DTPB model.

According to the analysis of the example, if only a traditional DTPB model is used to measure the impact of the variables, then the model does not consider that the variables will affect the original cause-and-effect relationships among the variables if they possess direct or indirect relationships. The original DTPB model variables cannot represent a complete set of relationships. A DEMATEL method was employed to reconstruct that DTPB model and, more importantly, to calculate reasonable DEMATEL threshold value. Thus, additional relationships of variables from the original DTPB model were obtained. For instance, the effects of A_2_ on A_1_ and A_12_, A_6_ on A_12_, A_7_ on A_12_, and A_11_ on A_1_ and A_6_, were obtained. These results can be helpful for the introduction of an information system such that the DTPB model not only calculates the behaviors and inclinations of employees in using food and beverage information systems, but also provides additional information regarding the information behavior use of dining service providers. This should contribute to the further introduction of food and beverage information systems and the improvement of food and beverage service quality.

Finally, one of the contributions of this paper is to propose a reconstructed DEMATEL threshold value method. Because this study presents a food and beverage information system as an example, this study is method-oriented; however, that implies that there are limits to the depth of this study’s inquiry into individual cases. Therefore, the proposed method should be extended to various fields of study in order to develop deeper and more practical implications.
